# Combined plasma syndecan-1 and renal resistive index as early predictors of sepsis-associated Acute kidney injury: a prospective observational study

**DOI:** 10.1080/0886022X.2026.2628387

**Published:** 2026-02-18

**Authors:** Rouxin Zhang, Danqing Zhang, Linxi Huang, Xiaohua Huang

**Affiliations:** a Department of Critical Care Medicine, The First Affiliated Hospital of Shantou University Medical College, Shantou, China; b Shantou University Medical College, Shantou, China; c Laboratory of Molecular Cardiology & Laboratory of Molecular Imaging, The First Affiliated Hospital of Shantou University Medical College, Shantou, China

**Keywords:** Sepsis associated acute kidney injury, endothelial glycocalyx, renal resistive index, bedside doppler ultrasound, macro- microcirculatory dysfunction

## Abstract

The interplay between macro-microcirculatory dysfunction and sepsis associated acute kidney injury (SA-AKI) remains elusive. This study aimed to explore the association between hemodynamics and endothelial damage in SA-AKI by evaluating the combined predictive value of renal resistive index (RRI) and plasma syndecan-1. This prospective observational study enrolled 80 septic patients admitted to the general intensive care unit (ICU) of a tertiary hospital from May to December 2024. Plasma syndecan-1 levels were measured at admission, and RRI was assessed within 24 h of ICU admission. Univariate and multivariate logistic regression models were applied to identify independent risk factors of SA-AKI. Diagnostic performance was evaluated using receiver operating characteristic (ROC) curve analysis by calculating the area under the curve (AUC). Among 80 septic patients, 41(51.25%) developed AKI. Syndecan-1 levels were significantly higher in the AKI group [109.95 (73.67–221.40) vs.73.67 (54.59–109.95) ng/mL, *p* = 0.007], and RRI values were markedly elevated (0.69 ± 0.08 vs. 0.60 ± 0.06, *p* < 0.001) compared to non-AKI patients. Univariate analysis revealed syndecan-1 (OR = 2.68, 95%CI 1.29–5.59) and RRI (OR = 1.18, 95%CI 1.09–1.28) as predictors of AKI. In multivariate models adjusted for confounders, both plasma syndecan-1 (OR = 3.57, 95%CI 1.01–12.64, *p* = 0.048) and RRI (OR = 1.19, 95% CI 1.07–1.33, *p* = 0.002) retained significance. A predictive model using a combination of plasma syndecan-1 and RRI achieved superior diagnostic performance (AUC 0.859, sensitivity 87.8%, specificity 92.3%). In patients with SA-AKI, elevated plasma syndecan-1 and RRI were identified as independent risk factors. The combination of syndecan-1 and RRI can serve as synergistic biomarkers for the prediction of SA-AKI.

## Introduction

Sepsis associated acute kidney injury (SA-AKI) is a significant challenge in critical care medicine. In the intensive care unit (ICU), the incidence of acute kidney injury (AKI) among sepsis patients ranges from 45% to 70%, with mortality rates exceeding 30% [1,2]. Beyond systemic alterations in renal perfusion, emerging evidence has revealed the importance of microcirculatory dysfunction and endothelial damage in the development of AKI [3–5]. The degradation of endothelial glycocalyx (eGCX) is a key pathophysiological element in sepsis, which is linked to AKI development [6]. Syndecan-1, a proteoglycan shed from the eGCX during injury, has emerged as a biomarker reflecting endothelial damage severity. Elevated syndecan-1 levels correlate with AKI incidence in sepsis [6,7]. However, its utility for early AKI prediction is yet to be explored.

While systemic hemodynamic instability drives macrocirculatory dysfunction, renal resistive index (RRI)—a quantitative measure of localized renal hemodynamic impairment—serves as a critical bridge linking systemic circulatory failure to organ-specific injury. Bedside Doppler Ultrasound is recognized as a noninvasive and repeatable diagnostic tool. Increasing evidence supports the capability of bedside ultrasound to accurately assess renal hemodynamics [8]. Studies have shown that the RRI measured by ultrasound can be used as a perfusion indicator of the renal circulation, and abnormal increases in RRI may be associated with the occurrence and severity of AKI [9]. Furthermore, studies have demonstrated that in cases of AKI, abnormalities in RRI occur before changes in serum creatinine (SCr) and can be used as an early diagnostic index for SA-AKI [10].

This synergy between hemodynamic alterations using RRI, and eGCX disruption using plasma syndecan-1 might offer complementary targets for early diagnosis. In our present study, we aimed to investigate the early diagnosis value of syndecan-1 and RRI in SA-AKI patients.

## Methods

### Patients and study design

This prospective cohort study was conducted in a 26-bed ICU at a tertiary university hospital in Shantou, China, from May to December 2024. The study was approved by the Ethics Committee for Clinical Research at the First Affiliated Hospital of Shantou University Medical College (B-2023-231). All procedures were conducted in accordance with the Declaration of Helsinki (as revised in 2013). Written informed consent was obtained from each patient or their next of kin.

**Figure 1.**
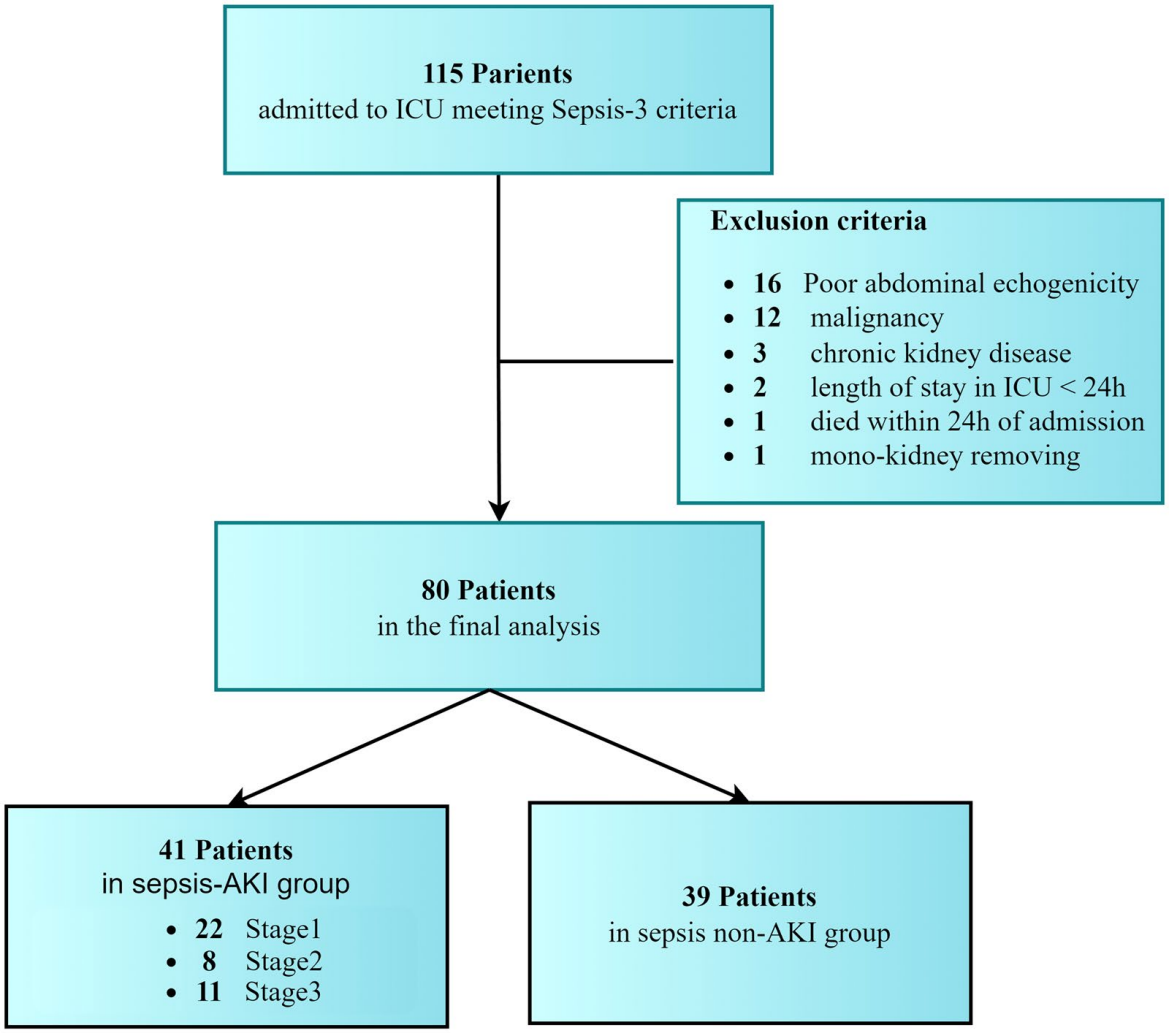
Flow-chart showing diagram of patients’ inclusion and exclusion. ICU: Intensive Care Unit, AKI: Acute Kidney Injury.

A total of 115 patients were enrolled in the preliminary screen. Inclusion criteria required adults (>18 years) diagnosed with sepsis and without prior ICU admission. Exclusion criteria comprised: (1) age <18 years; (2) ICU stay <24 h, or died within 24 h of admission; (3) pregnancy/lactation; (4) history of chronic kidney disease, end-stage renal disease, single functioning kidney, or kidney transplantation; (5) active malignancy, hematologic disorders, or autoimmune diseases; (6) poor abdominal ultrasonography conditions. After exclusions, 80 sepsis patients were stratified into two cohorts: sepsis without AKI (*n* = 39) and sepsis with AKI (*n* = 41). A detailed enrollment flowchart is provided in Figure 1.

### Definitions

AKI was diagnosed and staged according to the Kidney Disease: Improving Global Outcomes (KDIGO) 2012 criteria [11], defined by meeting at least one of the following: (1) SCr increase ≥26.5 μmol/L (≥0.3 mg/dL) within 48 h; (2) SCr ≥1.5 times baseline within the past 7 days (lowest value within 3–6 months pre-admission; admission SCr used if unavailable [12]; (3) Urine output ≤0.5 mL/kg/h for ≥6 h. Severity was classified into three stages based on SCr and urine output thresholds. Sepsis and septic shock were defined according to the 2016 American College of Chest Physicians/Society of Critical Care Medicine (ACCP/SCCM) Sepsis-3 criteria [13]. Sepsis required confirmed infection with a Sequential Organ Failure Assessment (SOFA) score ≥2 at ICU admission. Septic shock was defined as sepsis with persistent hypotension requiring vasopressors despite adequate fluid resuscitation.

### Data collected

Clinical data were prospectively collected using standardized case report forms. Key variables included demographic information, comorbidities, vital signs, laboratory parameters such as SCr, blood urea nitrogen (BUN), N-terminal pro-B-type natriuretic peptide (NT-proBNP), procalcitonin (PCT), serum cystatin C (sCys C) and homocysteine (HCY). Other physiological and clinical details were also collected, including the Acute Physiology and Chronic Health Evaluation II (APACHE II) score, SOFA score and septic shock status. Baseline and daily laboratory values were recorded during ICU admission, with primary analyses focusing on data from the first day. The outcome variables included duration of mechanical ventilation and continuous renal replacement therapy (CRRT) use during the ICU stay.

### Biomarker measurements

Plasma syndecan-1 was quantified as a biomarker of eGCX injury using a commercial ELISA kit (Human Syndecan-1 ELISA Kit, Abcam ab46506) with a detection range of 8–256 ng/mL and intra-assay variability of 6.2%. Whole blood samples (2 mL) were collected in heparinized tubes within 2 h of ICU admission. The samples were subsequently centrifuged at 3,000 rpm for 10 min, and the resulting plasma was labeled and stored at −20 °C. Additionally, plasma levels of Cys C, HCY, SCr and BUN were measured by the institutional clinical laboratory as part of routine admission testing.

### RRI measurements

RRI was measured using Doppler ultrasound (Mindray ME7 system, China) with a convex array transducer (C5-1 probe) to assess renal hemodynamics. All examinations were completed within 24 h of ICU admission by a single trained operator and supervised by a qualified ultrasound practitioner. The kidney was first identified *via* B-mode ultrasound to observe the parenchyma’s echogenicity. Subsequently, pulsed-wave Doppler mode was applied to visualize the renal artery, segmental artery, and interlobular artery. The interlobular artery was selected for pulsed Doppler ultrasound measurement. Three consecutive stable waveforms from the interlobular artery (predominantly right kidney) were captured. Upon obtaining at least three stable blood flow waveforms, the peak systolic velocity (PSV) and end-diastolic velocity (EDV) were measured, and their average was calculated to determine the mean RRI value of the right kidney. RRI was calculated using the following formula: RRI= (PSV − EDV)/PSV. Mean RRI values were derived from triplicate measurements.

### Statistical analysis

The target sample size for this study was determined *a priori* using the events per variable (EPV) criterion. A minimum EPV of 5–10 is generally recommended in the literature [14–16]. With six candidate predictor variables planned for inclusion in the model, a minimum of 49 participants would be required to meet an EPV of 5, whereas 97 participants would be required to achieve an EPV of 10. In this study, the final sample size was 80, which adequately met the requirement. Continuous variables were expressed as mean ± SD or median (interquartile range), as appropriate. Categorical variables were summarized as frequencies (%) and compared with Chi-square test or Fisher’s exact test. Syndecan-1 levels underwent log-transformation to approximate normality. When comparing between groups, the statistical test automatically selected for continuous variables was the Student t-test or Mann-Whitney U test. We evaluated the predictive value of syndecan-1 and RRI for the occurrence of AKI by constructing the receiver operating characteristic (ROC) curve and calculating the area under the ROC curve (AUC) with its 95% confidence interval. The Delong test was conducted to compare AUC values. Spearman’s rank correlation coefficient was employed to evaluate the relationship between biomarkers and AKI. Additionally, univariate and multivariate logistic regression analyses were performed to determine independent prognostic predictors.

All tests were two-tailed, and a *p* < 0.05 was considered statistically significant. All statistical tests were conducted using R version 4.3.1(R-project, Institute for Statistics and Mathematics, Vienna, Austria).

## Results

### Demographic and clinical characteristics of patients

A total of 115 septic patients were screened, with 80 patients meeting inclusion criteria (Figure 1). Based on Sepsis-3 and KDIGO 2012 criteria, 41 patients (51.25%) developed AKI, stratified as stage 1 (*n* = 22, 27.50%), stage 2 (*n* = 8, 10.00%), and stage 3 (*n* = 11, 13.75%).

The demographic and clinical characteristics were demonstrated in Table 1. The median age of the patients was 65 years (56, 72), with 33.80% (*n* = 27) of the cohort being female. Patients with AKI exhibited significantly elevated biomarkers such as oxygenation index (*p* = 0.019), lactic acid (*p* = 0.024), SCr (*p* < 0.001), BUN (*p* < 0.001), NT-proBNP (*p* = 0.001), sCys C (*p* < 0.001), HCY (*p* = 0.005) and PCT (*p* = 0.003) compared to the non-AKI group. Additionally, patients with AKI exhibited significantly higher APACHE II scores (*p* = 0.04) and SOFA scores (*p* = 0.039), and were more likely to require CRRT (*p* = 0.011) (Table 1). A more detailed version of this table was provided in Supplementary Table S1.

**Table 1.** Demographic and clinical characteristics for patients with sepsis.

Variables	Overall (*N* = 80)	Sepsis and non-AKI (*N* = 39)	Sepsis and AKI (*N* = 41)	*p*
Age	65 (56–72)	66 (59–72)	64 (49–70)	0.488
Sex				
Female	27 (33.8)	18 (46.2)	9 (22.0)	0.040
Male	53 (66.2)	21 (53.8)	32 (78.0)	
ICU Severity Scores				
APACHE II	17.00 (11.75–23.25)	15.00 (10.00–19.50)	19.00 (14.00–24.00)	0.040
SOFA	5.00 (3.75–8.00)	5.00 (3.00–7.50)	7.00 (4.00–9.00)	0.039
Co-morbidities				
Septic shock				
Yes	18 (22.5)	6 (15.4)	12 (29.3)	0.223
No	62 (77.5)	33 (84.6)	29 (70.7)	
Diabetes				
Yes	22 (27.5)	9 (23.1)	13 (31.7)	0.539
No	58 (72.5)	30 (76.9)	28 (68.3)	
Hypertension				
Yes	28 (35.0)	12 (30.8)	16 (39.0)	0.590
No	52 (65.0)	27 (69.2)	25 (61.0)	
Vital signs				
MAP, mmHg	83.00 (73.75–96.25)	83.00 (76.00–94.00)	85.00 (73.00–97.00)	0.870
Heart Rate, beats/min	96.75 ± 26.52	95.33 ± 22.76	98.10 ± 29.89	0.644
Oxygenation Index, mmHg	255.00 (187.25–320.00)	231.00 (181.50–265.50)	290.00 (193.00–408.00)	0.019
Laboratory parameters				
Lactic Acid, mmol/L	1.50 (1.00–2.40)	1.40 (0.85–1.90)	1.70 (1.20–2.70)	0.024
Creatinine, μmol/L	102.45 (66.00–158.12)	66.70 (59.25–92.35)	158.10 (125.20–195.00)	<0.001
NT-proBNP, pg/mL	687.90 (315.58–1781.25)	494.20 (256.70–834.15)	1207.00 (500.10–4930.00)	0.001
BUN, mmol/L	8.35 (6.47–11.43)	6.50 (4.40–8.35)	10.50 (8.30–17.30)	<0.001
Cystatin C, mg/L	1.20 (0.98–1.70)	1.00 (0.80–1.10)	1.60 (1.20–2.10)	<0.001
Homocysteine, μmol/L	11.80 (7.00–13.95)	9.50 (6.70–13.00)	13.20 (9.80–16.30)	0.005
Procalcitonin	2.34 (0.37–14.80)	1.06 (0.20–5.42)	6.50 (0.71–27.50)	0.003
CRRT				
Yes	9 (11.2)	0 (0.0)	9 (22.0)	0.011
No	71 (88.8)	39 (100.0)	32 (78.0)	
Mechanical Ventilation				
Yes	46 (57.5)	21 (53.8)	25 (61.0)	0.676
No	34 (42.5)	18 (46.2)	16 (39.0)	
RRI	0.65 ± 0.08	0.60 ± 0.06	0.69 ± 0.08	<0.001
Syndecan-1, ng/ml	90.25(65.95-190.73)	73.67(54.59–109.95)	109.95(73.67–221.40)	0.006

Continuous variables were expressed as mean ± standard deviation, or median (interquartile range, IQR), as appropriate.Categorical variables were presented as n (%).

*APACHE* Acute Physiology and Chronic Health Evaluation, *SOFA* Sepsis-related Organ Failure Assessment score, *MAP* Mean Arterial Pressure, *NT-proBNP* N-terminal pro-B-type natriuretic peptide, *BUN* Blood Urea Nitrogen, *CRRT* Continuous Renal Replacement Therapy, *RRI* Renal Resistive Index.

### Association of RRI, plasma syndecan-1 and AKI

**Figure 2.**
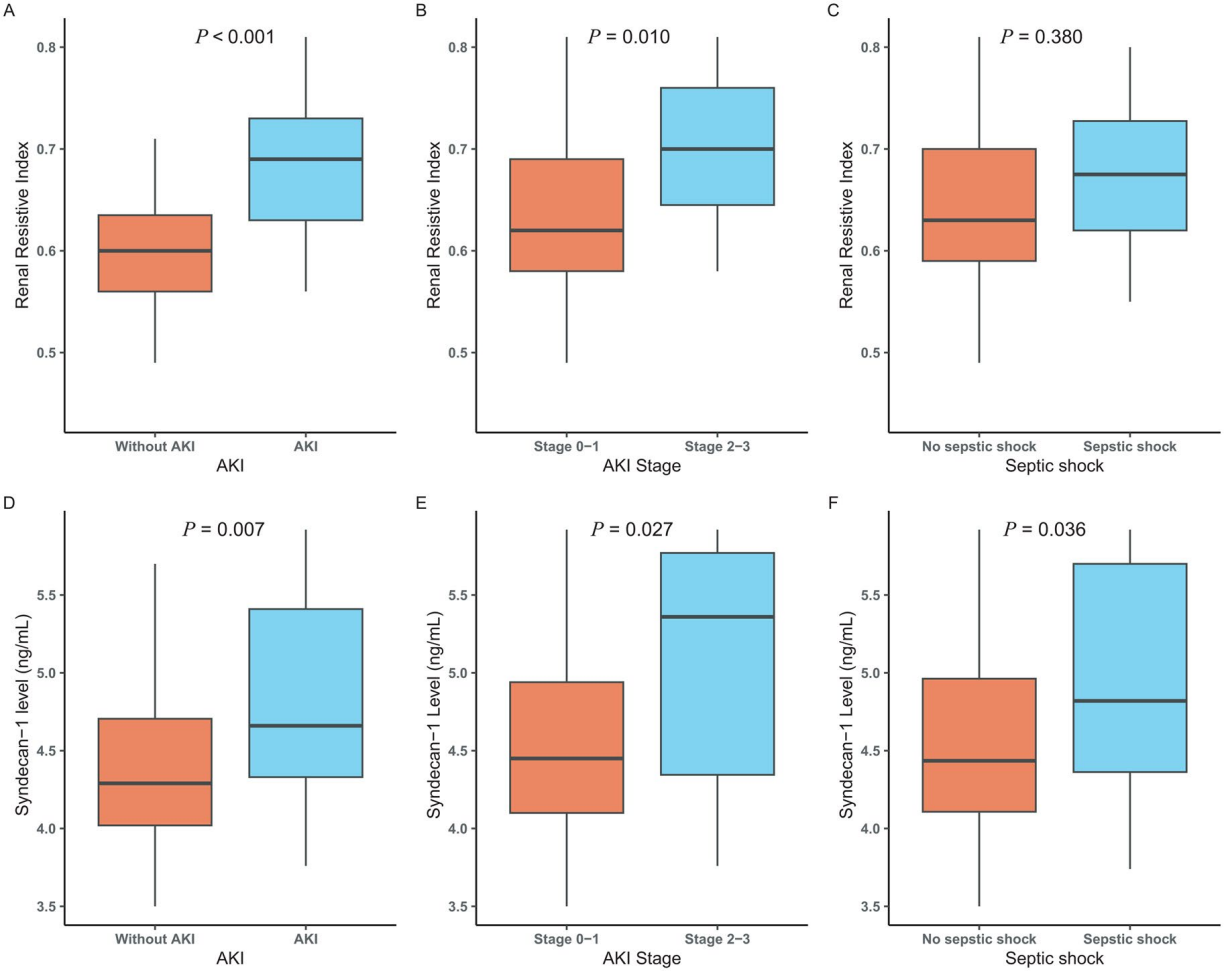
Boxplot of syndecan-1 and renal resistive index according to presence of AKI and septic shock. Boxplots (A, B, C) show the distribution of the renal resistive index (RRI) across AKI status, AKI stage, and septic shock. Boxplots (D, E, F) show the distribution of syndecan-1 levels across the same groups. *p* -values of syndecan-1 and RRI were determined using Mann–Whitney U Test and t.test. *p* < 0.05 were considered statistically significant.

Our study found that elevated plasma syndecan-1 level and RRI were independently associated with AKI in septic patients. Plasma syndecan-1 levels on ICU admission were notably higher in the AKI group compared to the non-AKI group [109.95 (73.67–221.40) vs. 73.67 (54.59–109.95) ng/ml, *p* = 0.007]. RRI was also significantly elevated in the AKI group compared to the non-AKI group (0.69 ± 0.08 vs. 0.60 ± 0.06, *p* < 0.001). (Table 1, Figure 2A, 2D).

Furthermore, our study demonstrated that elevated plasma syndecan-1 level and RRI were positively correlated with AKI severity (*p* < 0.05). Patients with AKI stages 2–3 exhibited markedly higher syndecan-1 levels compared to those with AKI stages 0–1 [213.29 (77.72–321.45) vs. 85.71 (60.34–139.34) ng/mL, *p* = 0.027]. Similarly, the RRI was significantly increased in the AKI stage 2–3 group compared to the stage 0–1 group (0.70 ± 0.09 vs. 0.63 ± 0.07, *p* = 0.01) (Table S2, Figure 2B, 2E).

In addition to comparisons between AKI and non-AKI patients, we further analyzed biomarker differences in patients with or without shock. Patients with septic shock exhibited significantly higher syndecan-1 levels compared to those without shock [109.95 (73.67–221.40) vs. 73.67 (54.59–109.95) ng/mL, *p* = 0.036]. Other relevant variables in the septic shock group, including albumin, PCT, and CRP, also demonstrated higher levels (*p* < 0.05) (Table 2, as details in Table S2). Notably, RRI showed no significant difference between shock and non-shock groups (0.66 ± 0.09 vs. 0.64 ± 0.08; *p* = 0.357), indicating its specificity for AKI-related hemodynamic alterations rather than a global circulatory failure.

**Table 2.** Comparison characteristics and biomarker levels in patients with and without septic shock.

Variables	Overall (*N* = 80)	Non-septic shock (*N* = 62)	Septic shock (*N* = 18)	*p*
Age	65 (56–72)	65 (56–72)	66 (56–70)	0.809
Sex				
Female	27 (33.8)	19 (30.6)	8 (44.4)	0.420
Male	53 (66.2)	43 (69.4)	10 (55.6)	
Lactic Acid, mmol/L	1.50 (1.00–2.40)	1.50 (0.92–2.20)	1.80 (1.35–2.63)	0.114
Creatinine, μmol/L	102.45 (66.00–158.12)	102.45 (63.30–157.67)	101.80 (69.07–154.62)	0.991
NT-proBNP, pg/mL	687.90 (315.58–1781.25)	653.15 (287.75–1581.00)	786.10 (431.92–4133.50)	0.276
Albumin, μmol/L	29.68 ± 6.24	30.68 ± 6.12	26.26 ± 5.53	0.007
Total Bilirubin, μmol/L	15.50 (9.88–24.30)	16.10 (11.05–24.53)	12.95 (7.95–22.28)	0.261
BUN, mmol/L	8.35 (6.47–11.43)	8.30 (5.85–12.13)	8.75 (7.08–9.82)	0.931
Cystatin C, mg/L	1.20 (0.98–1.70)	1.25 (0.90–1.70)	1.20 (1.00–1.60)	0.822
Homocysteine, μmol/L	11.80 (7.00–13.95)	11.90 (7.75–14.05)	11.15 (6.18–12.82)	0.372
Procalcitonin	2.34 (0.37–14.80)	1.15 (0.28–7.13)	27.00 (4.03–48.38)	<0.001
C-Reactive Protein, mg/L	69.80 (28.87–113.25)	58.75 (21.35–107.30)	96.15 (71.33–136.32)	0.011
IL-6, pg/mL	547.50 (109.25–1877.25)	219.50 (89.40–1162.75)	2702.00 (612.38–4000.00)	0.002
RRI	0.65 ± 0.08	0.64 ± 0.08	0.66 ± 0.09	0.357
Syndecan-1, ng/ml	90.25(65.95-190.73)	85.71(60.34-143.94)	126.61(77.72–300.41)	0.039

Continuous variables were expressed as mean ± standard deviation, or median (interquartile range, IQR), as appropriate.Categorical variables were presented as n (%).

*NT-proBNP* N-terminal pro-B-type natriuretic peptide, *BUN* Blood Urea Nitrogen, *IL-6* Interleukin-6, *RRI* Renal Resistive Index.

### Univariate and multivariate logistic regression analysis in AKI

**Figure 3.**
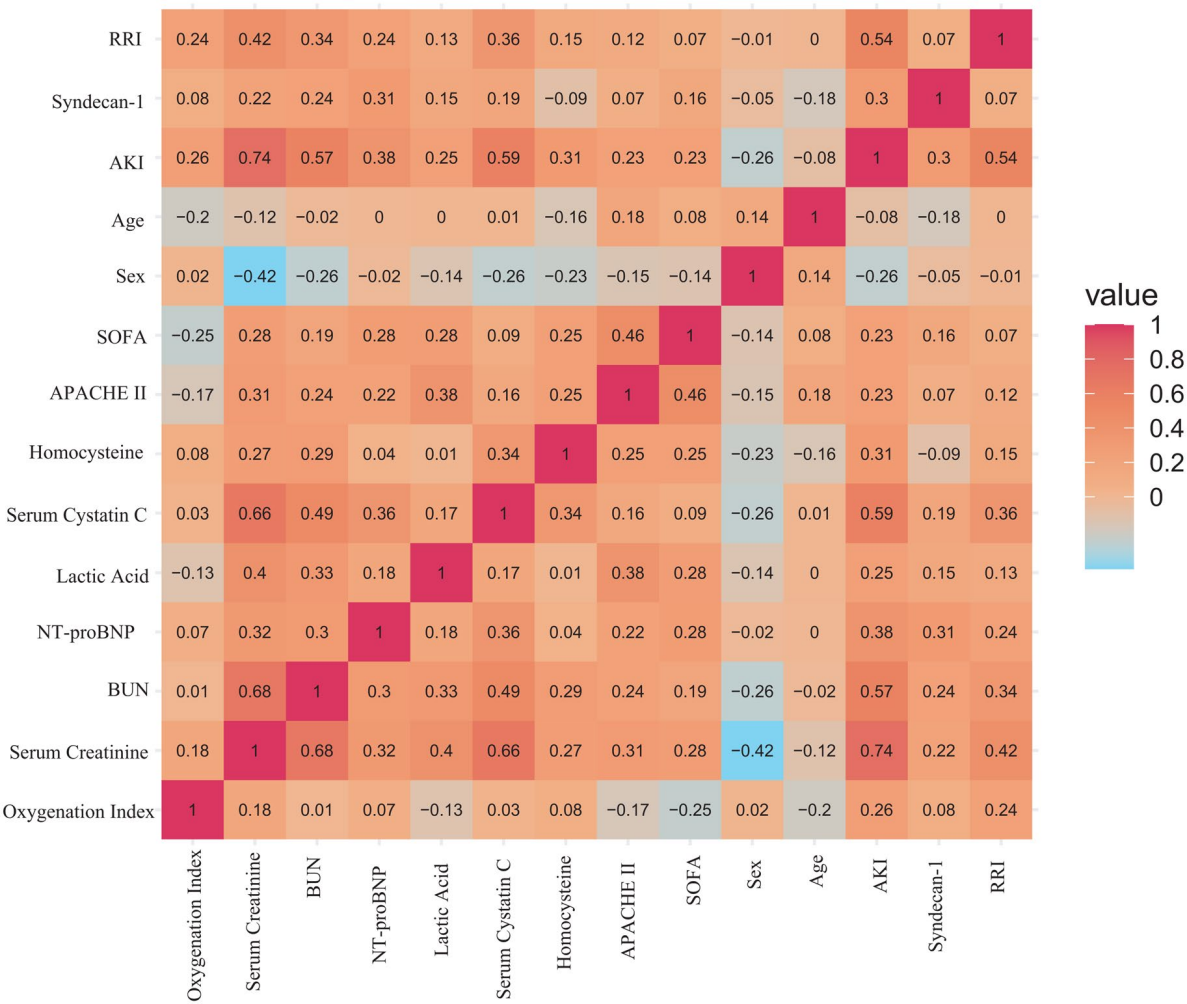
The correlation heatmap represents the correlation (Spearman) among biomarkers and AKI. The color-coded scale of correlation is shown on the right, where a red color indicates a positive correlation, while a green color indicates a negative correlation. The color intensity corresponds to correlation coefficient magnitude, and the values represent the Spearman correlation coefficients.

In the univariate logistic regression analysis, several biomarkers and clinical indicators were found to be significantly associated with the occurrence of AKI. Specifically, elevated levels of syndecan-1 and RRI showed strong associations with AKI, with odds ratios (OR) of 2.68 (95% CI: 1.29–5.59, *p* = 0.008) and 1.18 (95% CI 1.09–1.28, *p* = 0.001), respectively. Additional independent risk factors for AKI included male sex (OR = 3.05, 95% CI: 1.15–8.05, *p* = 0.024), lactic acid (OR = 1.87, 95% CI: 1.12–3.14, *p* = 0.017), oxygenation index (OR = 1.01, 95% CI: 1.00–1.01, *p* = 0.019), Cys C(OR = 4.90, 95% CI: 1.82–13.16, *p* = 0.002), HCY (OR = 1.17, 95% CI: 1.05–1.30, *p* = 0.005), NT-proBNP (OR = 1.56, 95% CI: 1.12–2.17, *p* = 0.009), APACHE II score (OR = 1.06, 95% CI: 1.00–1.13, *p* = 0.045), and SOFA score (OR = 1.18, 95% CI: 1.01–1.38, *p* = 0.041) (Table 3). To further explore the relationships among these biomarkers and AKI, we calculated Spearman correlation coefficients and visualized in a heatmap. In this heatmap, positive correlations were represented in red, while negative correlations were shown in green (Figure 3).

**Table 3.** Univariate and multivariate logistic regression analysis for factors linked to patients with AKI.

	Univariate			Multivariate	
	Odds Ratio (95% CI)	*P*		Odds Ratio (95% CI)	*p*
Syndecan-1	2.68 (1.29–5.59)	0.008		3.57 (1.01–12.64)	0.048
Renal Resistive Index*	1.18 (1.09–1.28)	0.001		1.19 (1.07–1.33)	0.002
Age, years	0.98 (0.96–1.01)	0.320			
Male, sex	3.05 (1.15–8.05)	0.024		4.75 (0.74–30.36)	0.100
Lactic Acid, mmol/L	1.87 (1.12–3.14)	0.017		1.82 (0.80–4.14)	0.155
Oxygenation Index	1.01 (1.00–1.01)	0.019		1.01 (1.00–1.02)	0.094
Cystatin C, mg/L	4.90 (1.82–13.16)	0.002		1.98 (0.80–4.92)	0.140
Homocysteine, μmol/L	1.17 (1.05–1.30)	0.005		1.16 (1.00–1.35)	0.046
APACHE II	1.06 (1.00–1.13)	0.045		1.01 (0.89–1.14)	0.870
SOFA	1.18 (1.01–1.38)	0.041		1.05 (0.79–1.40)	0.730
NT-proBNP, pg/mL**	1.56 (1.12–2.17)	0.009			

*APACHE* Acute Physiology and Chronic Health Evaluation, *SOFA* Sepsis-related Organ Failure Assessment score, *RRI* Renal Resistive Index, *NT-proBNP* N-terminal pro-B-type natriuretic peptide, *CI* Confidence interval.

*RRI was presented as a percentage (%). ** NT-proBNP was log-transformed due to its large OR value for easier computation.

Furthermore, a multivariate logistic regression analysis was conducted to identify independent predictors of AKI. To ensure model stability and avoid multicollinearity, we included only variables that were statistically significant (*p* < 0.05) in the univariate logistic regression and had a correlation coefficient with syndecan-1 of less than 0.3. The analysis revealed that syndecan-1 (OR = 3.57, 95% CI 1.01–12.64, *p* = 0.048), RRI (OR = 1.19, 95% CI 1.07–1.33, *p* = 0.002), and HCY (OR = 1.16, 95% CI 1.00–1.35, *p* = 0.046) were independent risk factors and early predictors in AKI (Table 3).

### Diagnostic model construction and performance evaluation

**Figure 4.**
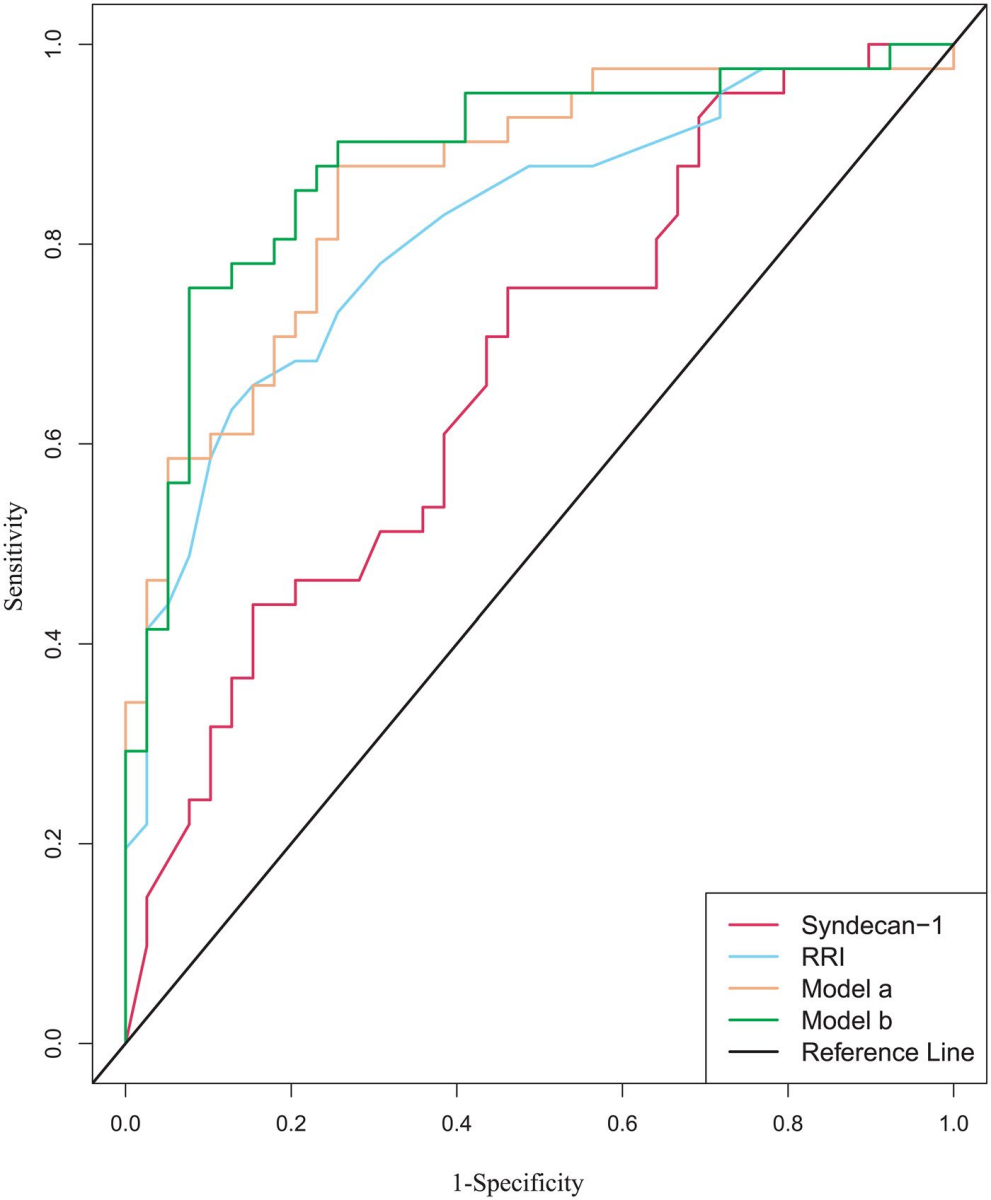
The ROC curve displays the ability of RRI, syndecan-1 and other Models for AKI patients. The predictive performance of individual biomarkers and combination models was shown: syndecan-1 (red curve; AUC = 0.680), RRI (blue curve; AUC = 0.813), Model A (orange curve; AUC = 0.859), and Model B (green curve; AUC = 0.885). Pairwise comparison of ROC curves was performed using DeLong’s test: Model A vs. syndecan-1, *p* = 0.0021; Model A vs. RRI, *p* = 0.15. RRI: Renal Resistive Index, Model A: RRI + Syndecan-1, Model B: Syndecan-1 + RRI + age + sex + diabetes + hypertension.

In order to examine the influence of demographic and clinical variables on AKI, two diagnostic models were constructed. Model A comprised syndecan-1 and RRI with mutual adjustment. Model B was further adjusted for age, sex, diabetes, and hypertension. In both models, the statistically significant independent variables identified in multivariate analysis remained consistent (Table 4). The diagnostic efficacy of all independent variables and models was evaluated using ROC curve analysis, with the results presented in Table 5 and Figure 4.

**Table 4.** The relationship between syndecan-1 and RRI and AKI in patients with sepsis.

Variables	Unadjusted OR (95% CI)	*p*	Model a OR (95% CI)	*p*	Model b OR (95% CI)	*p*
Syndecan-1	2.68 (1.29–5.59)	0.008	3.62 (1.42–9.19)	0.007	3.78 (1.40–10.23)	0.009
RRI	1.18 (1.09–1.28)	<0.001	1.21 (1.10–1.32)	<0.001	1.22 (1.11–1.34)	<0.001

*RRI* Renal Resistive Index, *OR* Odds Ratio, *CI* Confidence interval.

Model A: Syndecan-1(adjusted for RRI). RRI (adjusted for syndecan-1).

Model B: Adjusted for age, sex, diabetes, hypertension.

**Table 5.** Comparison of the predictive performance of RRI, syndecan-1, homocysteine and other models for AKI patients.

Variables	AUC	95%CI	Sensitivity	Specificity
RRI	0.813	0.719 − 0.908	63.4%	87.2%
Syndecan-1, ng/mL	0.680	0.557 − 0.792	75.6%	53.8%
Homocysteine, μmol/mL	0.680	0.562 − 0.799	46.3%	89.7%
Syndecan-1+ Homocysteine	0.743	0.635 − 0.851	51.2%	89.7%
RRI+ Homocysteine	0.850	0.764 − 0.936	70.7%	92.3%
Model A	0.859	0.778 − 0.941	87.8%	74.4%
Model B	0.885	0.809 − 0.961	75.6%	92.3%

*RRI* Renal Resistive Index, *AUC* Area under the curve, *CI* Confidence interval.

Model A: RRI + Syndecan-1.

Model B: Syndecan-1 + RRI+age + sex + diabetes + hypertension.

The findings demonstrated that Model B, adjusted for all confounders, achieved an AUC of 0.885, sensitivity of 75.6% and specificity of 92.3%. The AUCs for syndecan-1 and RRI were 0.680 and 0.813, respectively, with an optimal cutoff point of 75.82 ng/ml (sensitivity 75.6%, specificity 53.8%) for syndecan-1 and 0.68 (sensitivity 63.4%, specificity 87.2%) for RRI. Notably, the combination of syndecan-1 and RRI (model A) achieved an AUC of 0.859 (sensitivity 87.8%, specificity 74.4%), indicating superior predictive performance compared to each variable alone. DeLong’s test revealed that the model A (syndecan-1 + RRI) significantly outperformed syndecan-1 alone (*p* = 0.0021), but there were no significant differences in RRI alone (*p* = 0.15).

## Discussion

In this prospective study, we identified plasma syndecan-1 and RRI as novel, independent predictors and early diagnostic markers of SA-AKI. Both biomarkers retained robust predictive value for SA-AKI even after adjustment for clinical confounders. Notably, both biomarkers correlated with AKI severity. The combined use of these biomarkers significantly improved diagnostic accuracy compared to individual markers alone, demonstrating the synergistic value for early SA-AKI detection.

The early hypothesis posited that SA-AKI originated from pre-renal hypoperfusion. However, clinical observations revealed that not all AKI patients exhibited concomitant hypoperfusion. Paradoxically, early animal studies by Ravikant et al. demonstrated that porcine models of sepsis displayed AKI despite an increased renal blood flow [17]. More recently, a retrospective observational study by Legrand et al. further indicated that SA-AKI may be more closely related to renal microcirculatory dysfunction and endothelial damage rather than solely to systemic renal hypoperfusion [18]. To further elucidate the impact of local hemodynamic response on kidney function, we employed bedside Doppler ultrasound to measure the RRI [19]. An elevation in RRI generally indicated an increase in renal artery resistance, indicative of alterations in renal local circulation. Researches demonstrated that RRI increase in sepsis was correlated with systemic hemodynamic disturbances, renal perfusion insufficiency, and damage to renal vessels [20–22]. Shen et al. have recently reported that RRI can function as an indicator of renal perfusion, with its abnormal elevation potentially linked to the occurrence and severity of AKI. Furthermore, its progressive increase (ΔRRI ≥0.05) is associated with prolonged renal recovery [9]. In a prospective study, Lerolle et al. [20] suggested that elevated RRI measured within 24 h of ICU admission predicts the AKI development within 5 days. Patients with AKI were shown to have a significantly elevation in RRI, and high RRI was an independent risk factor for SA-AKI [10]. Taken together, RRI can be a real-time tool to detect early perfusion deficits in sepsis.

Consistent with these findings, our study demonstrated that RRI is independently associated with SA-AKI, with an optimal cutoff value of 0.68 and an AUC of 0.813 (95% CI: 0.719–0.908). Elevated RRI values reflect hemodynamic derangements characterized by increased intrarenal vascular resistance and impaired autoregulation. This ultimately results in diminished renal perfusion and the development of AKI [23,24]. In a prospective cohort study, Haitsma Mulier et al. found that RRI could significantly predict the occurrence of AKI in sepsis patients at admission, especially in patients with AKI stages 2–3, where its predictive ability was more pronounced [25]. Aligned with their findings, our data further validated a severity-dependent pattern of RRI, demonstrating a graded RRI elevation across AKI stages. In sum, RRI can be used as a dynamic biomarker for AKI severity stratification.

Although RRI can reflect renal cortical blood flow resistance [9], the interplay of factors such as renal microcirculation perfusion, inflammatory responses, and cellular metabolic disturbances in septic shock may obscure changes in RRI. Its integration with microcirculatory biomarkers might substantially improve the predictive performance. To evaluate microcirculatory function, syndecan-1 was used as a sensitive biomarker of abnormal degradation of eGCX [26]. The association between elevated syndecan-1 levels and sepsis prognosis, as discussed in our previous review [27], further validates our ongoing investigation into this biomarker. The eGCX is a critical component of the vascular endothelial barrier regulating permeability and inflammation [7]. In sepsis, exposure to inflammatory mediators disrupts eGCX integrity, which might contribute to microcirculatory disturbances [28]. This loss facilitates the adhesion of leukocytes and platelets to the damaged eGCX [29], exacerbating the inflammatory response, promoting local blood stasis, and ultimately leading to microcirculatory thrombosis. These events result in microcirculatory disturbances and organ dysfunction, including AKI [26,29]. In our study, we observed that RRI values did not significantly differ between sepsis patients with shock and those without shock. However, syndecan-1 levels were significantly higher in patients with septic shock. This may suggest the macro-micro mismatch and a microcirculatory dysfunction as one of the potential mechanisms underlying SA-AKI.

Piotti et al. reported that a strong association between elevated syndecan-1 levels and organ dysfunction as well as mortality in severe sepsis or septic shock patients in the ICU [30]. In children who underwent cardiac surgery, an early increased postoperative syndecan-1 level displays a high correlation with AKI incidence [31]. Similarly, Puskarich et al. demonstrated that syndecan-1 levels were markedly elevated in sepsis patients, and among those with severe sepsis or septic shock, plasma syndecan-1 levels were significantly higher in AKI compared to non-AKI patients [32]. However, current evidence presents mixed findings, with certain studies failing to show an independent correlation between elevated syndecan-1 and AKI incidence after multivariate adjustment [33]. This discrepancy with our findings may be attributed to heterogeneity in the study populations, suggesting that the predictive value of syndecan-1 for AKI may vary across different research contexts.

Our findings demonstrated that syndecan-1 was independently associated with AKI, with an optimal cutoff value of 75.82 ng/ml, yielding an AUC of 0.680 (95% CI: 0.557–0.792), and sensitivity and specificity rates of 75.6% and 53.7%, respectively. Additionally, the elevation of syndecan-1 level was correlated with AKI severity. These findings highlight the potential of syndecan-1 as a biomarker for AKI risk stratification in sepsis patients. While the moderate specificity suggests potential utility as a screening tool, clinical application would require confirmation in external cohorts. In contrast, RRI demonstrated inverse performance characteristics: moderate sensitivity (63.4%) but high specificity (87.2%), highlighting complementary diagnostic strengths between endothelial injury and hemodynamic biomarkers. Notably, integrating these markers into a composite model significantly enhanced predictive accuracy, achieved an AUC of 0.859 (95% CI: 0.778–0.941; sensitivity = 87.8%, specificity = 74.4%), outperforming individual markers. This improvement was statistically validated by DeLong’s test, which revealed that the Model A (syndecan-1 + RRI) significantly surpassed the performance of syndecan-1 alone (*p* = 0.0021). However, no significant difference was found when compared to RRI alone (*p* = 0.15). This discrepancy may be attributed to the pathophysiological heterogeneity of intrarenal blood flow distribution in sepsis. In an animal study on sepsis, Legrand et al. demonstrated that alterations in endothelial dysfunction and microcirculation contributed to increased variability in renal blood flow [34]. This finding aligned with clinical observations obtained through orthogonal polarization spectral (OPS) imaging, which have directly visualized significant microcirculatory heterogeneity in septic patients. This heterogeneity is characterized by the concurrent presence of non-perfused or hypoperfused capillaries alongside normally perfused vessels [35]. Additionally, Legrand et al. found that systemic hemodynamic parameters such as MAP and cardiac output (CO) did not significantly differ between AKI and non-AKI groups in SA-AKI patients [18]. Therefore, the observed discrepancies between syndecan-1 and RRI in certain patients highlight the heterogeneity and complexity of SA-AKI. Rather than diminishing their utility, these inconsistencies underscored the importance of a multi-dimensional biomarker approach. Taken together, the combined use of macro- and micor-biomarkers enhances early SA-AKI detection, enabling clinicians to initiate targeted therapies.

## Limitations and future directions

Our study is subject to several limitations. Firstly, the relatively small sample size and single-center, prospective design may limit the generalizability of our findings and introduce selection bias. Future research should aim to increase the sample size and adopt a multicenter approach to enhance the robustness and applicability of the results. Secondly, although the statistical models accounted for multiple confounding factors, other potential influences, such as the use of nephrotoxic drugs, fluid management strategies and specific etiologies of sepsis were not fully addressed due to limitations in data granularity. These aspects remain significant and should be explored in subsequent studies. Thirdly, while syndecan-1 and the RRI were identified as independent risk factors for SA-AKI, their precise mechanisms and pathophysiological roles in SA-AKI are not yet fully understood and require further investigations. Addressing these limitations in future research will be crucial for advancing our understanding and improving the clinical management of SA-AKI.

## Conclusion

Our study indicates that syndecan-1 and RRI are robust biomarkers for predicting SA-AKI. Syndecan-1, a marker of eGCX degradation, and RRI, a measure of renal hemodynamic status, each independently predict SA-AKI and correlate strongly with the severity of SA-AKI. When used in combination, these biomarkers significantly enhance diagnostic sensitivity and specificity compared to their individual application. Our findings suggest that integrating syndecan-1 and RRI into clinical algorithms could bridge the gap between endothelial injury and hemodynamic dysfunction, thereby improving early diagnosis. This integrated approach has the potential to provide a tool for the early identification of high-risk patients, enabling more timely interventions and ultimately improving outcomes for septic patients.

## Supplementary Material

Supplementary_Table_S2_clean.docx

Supplementary_Table_S1_clean.docx

## Data Availability

The datasets used and/or analyzed during the current study are available from the corresponding author on reasonable request.
