# Supplementary material for: Combined plasma syndecan-1 and renal resistive index as early predictors of sepsis-associated Acute kidney injury: a prospective observational study
Source: Ren Fail. 2026 Feb 18;48(1):2628387. doi: 10.1080/0886022X.2026.2628387 (PMC12918287; doi:10.1080/0886022X.2026.2628387)
Supplement: Supplementary_Table_S2_clean.docx [file IRNF_A_2628387_SM7422.docx]

**Table S2 Comparison characteristics and biomarker levels in patients with and without septic shock**

| Variables | Overall (N = 80) | Non-septic shock  (N = 62) | Septic shock  (N = 18) | *P* |
| --- | --- | --- | --- | --- |
| Age | 65 (56-72) | 65 (56-72) | 66 (56-70) | 0.809 |
| Sex |  |  |  |  |
| Female | 27 (33.8) | 19 (30.6) | 8 (44.4) | 0.420 |
| Male | 53 (66.2) | 43 (69.4) | 10 (55.6) |  |
| ICU Severity Scores |  |  |  |  |
| APACHE II | 17.00 (11.75-23.25) | 17.00 (12.00-21.00) | 19.50 (11.50-25.50) | 0.464 |
| SOFA | 5.00 (3.75-8.00) | 5.00 (3.00-8.00) | 5.50 (5.00-7.00) | 0.194 |
| Co-morbidities |  |  |  |  |
| AKI |  |  |  |  |
| Yes | 41 (51.2) | 29 (46.8) | 12 (66.7) | 0.223 |
| No | 39 (48.8) | 33 (53.2) | 6 (33.3) |  |
| AKI Stage |  |  |  |  |
| Yes | 22 (27.5) | 17 (27.4) | 5 (27.8) | 0.214 |
| No | 39 (48.8) | 33 (53.2) | 6 (33.3) |  |
| Diabetes |  |  |  |  |
| Yes | 58 (72.5) | 43 (69.4) | 15 (83.3) | 0.385 |
| No | 22 (27.5) | 19 (30.6) | 3 (16.7) |  |
| Hypertension |  |  |  |  |
| Yes | 28 (35.0) | 25 (40.3) | 3 (16.7) | 0.116 |
| No | 52 (65.0) | 37 (59.7) | 15 (83.3) |  |
| Pneumonia |  |  |  |  |
| Yes | 44 (55.0) | 34 (54.8) | 10 (55.6) | 1.000 |
| No | 36 (45.0) | 28 (45.2) | 8 (44.4) |  |
| Perforated Peptic Ulcer |  |  |  |  |
| Yes | 17 (21.2) | 12 (19.4) | 5 (27.8) | 0.659 |
| No | 63 (78.8) | 50 (80.6) | 13 (72.2) |  |
| Atherosclerosis |  |  |  |  |
| Yes | 13 (16.2) | 12 (19.4) | 1 (5.6) | 0.301 |
| No | 67 (83.8) | 50 (80.6) | 17 (94.4) |  |
| Congestive Heart Failure |  |  |  |  |
| Yes | 13 (16.2) | 5 (8.1) | 8 (44.4) | 0.001 |
| No | 67 (83.8) | 57 (91.9) | 10 (55.6) |  |
| Vital signs |  |  |  |  |
| MAP, mmHg | 83.00 (73.75-96.25) | 85.00 (75.50-99.00) | 80.50 (70.75-90.75) | 0.112 |
| Heart Rate, beats/min | 96.75±26.52 | 95.52±27.35 | 101.00±23.66 | 0.443 |
| Oxygenation Index, mmHg | 255 (187.25-320.00) | 250 (185.75-304.75) | 275 (198.00-366.50) | 0.454 |
| Laboratory parameters |  |  |  |  |
| Serum Glucose, mg/dL | 8.30 (6.68-10.75) | 8.20 (6.43-10.50) | 9.40 (7.58-12.38) | 0.112 |
| Lactic Acid, mmol/L | 1.50 (1.00-2.40) | 1.50 (0.92-2.20) | 1.80 (1.35-2.63) | 0.144 |
| Creatinine, μmol/L | 102.45 (66.00-158.12) | 102.45 (63.30-157.67) | 101.80 (69.07-154.62) | 0.991 |
| NT-proBNP , pg/mL | 687.90 (315.58-1781.25) | 653.15 (287.75-1581.00) | 786.10 (431.92-4133.50) | 0.276 |
| Albumin, umol/L | 29.68±6.24 | 30.68±6.12 | 26.26±5.53 | 0.007 |
| Total Bilirubin, umol/L | 15.50 (9.88-24.30) | 16.10 (11.05-24.53) | 12.95 (7.95-22.28) | 0.261 |
| BUN, mmol/L | 8.35 (6.47-11.43) | 8.30 (5.85-12.13) | 8.75 (7.08-9.82) | <0.931 |
| Cystatin C, mg/L | 1.20 (0.98-1.70) | 1.25 (0.90-1.70) | 1.20 (1.00-1.60) | <0.822 |
| Homocysteine, μmol/L | 11.80 (7.00-13.95) | 11.90 (7.75-14.05) | 11.15 (6.18-12.82) | 0.372 |
| Prothrombin Time, s | 13.10 (12.20-14.43) | 13.00 (12.03-13.70) | 14.20 (13.00-15.90) | 0.012 |
| APTT, s | 29.20 (26.73-34.05) | 29.00 (25.92-33.60) | 33.10 (28.90-35.80) | 0.029 |
| Serum D-Dimer, mg/L | 4815.00 (1607.50-8900.00) | 4815.00 (1582.50-9275.00) | 4465.00 (2057.50-6982.50) | 0.945 |
| White Blood Cell, 10^9^/L | 11.77±5.86 | 12.25±5.40 | 10.09±7.13 | 0.170 |
| Lymphocytes, 10^9^/L | 0.80 (0.60-1.20) | 0.80 (0.60-1.10) | 0.85 (0.60-1.37) | 0.795 |
| Platelets, 10^9^/L | 183.50 (137.50-255.50) | 185.50 (146.25-253.25) | 182.00 (132.25-246.75) | 0.954 |
| Serum Procalcitonin | 2.34 (0.37-14.80) | 1.15 (0.28-7.13) | 27.00 (4.03-48.38) | <0.001 |
| C-Reactive Protein, mg/L | 69.80 (28.87-113.25) | 58.75 (21.35-107.30) | 96.15 (71.33-136.32) | 0.011 |
| IL-6, pg/mL | 547.50 (109.25-1877.25) | 219.50 (89.40-1162.75) | 2702.00 (612.38-4000.00) | 0.002 |
| Serum Amyloid A, mg/L | 141.00 (81.32-150.00) | 141.00 (67.50-150.00) | 150.00 (93.22-150.00) | 0.310 |
| Norepinephrine |  |  |  |  |
| Yes | 27 (33.8) | 13 (21.0) | 14 (77.8) | <0.001 |
| No | 53 (66.2) | 49 (79.0) | 4 (22.2) |  |
| Dopamine |  |  |  |  |
| Yes | 18 (22.5) | 14 (22.6) | 4 (22.2) | 1.000 |
| No | 62 (77.5) | 48 (77.4) | 14 (77.8) |  |
| CRRT |  |  |  |  |
| Yes | 9 (11.2) | 5 (8.1) | 4 (22.2) | 0.211 |
| No | 71 (88.8) | 57 (91.9) | 14 (77.8) |  |
| Mechanical Ventilation |  |  |  |  |
| Yes | 46 (57.5) | 33 (53.2) | 13 (72.2) | 0.244 |
| No | 34 (42.5) | 29 (46.8) | 5 (27.8) |  |
| Hospital LOS, days | 16.60 (10.52-30.93) | 15.00 (9.93-30.32) | 23.40 (12.82-32.35) | 0.156 |
| ICU LOS, days | 4.85 (2.80-11.75) | 4.80 (2.73-10.35) | 5.75 (3.42-12.15) | 0.348 |
| RRI | 0.65±0.08 | 0.64±0.08 | 0.66±0.09 | 0.357 |
| Syndecan-1, ng/ml | 90.25（65.95-190.73） | 85.71（60.34-143.94） | 126.61（77.72-300.41） | 0.039 |

Continuous variables were expressed as mean ± standard deviation, or median (interquartile range, IQR), as appropriate. Categorical variables were presented as n (%).

**Abbreviations：**AKI: Acute Kidney Injury; APACHE: Acute Physiology and Chronic Health Evaluation; SOFA: Sepsis-related Organ Failure Assessment score; MAP: Mean arterial pressure; NT-proBNP: N-terminal pro-B-type natriuretic peptide; BUN: Blood Urea Nitrogen; APTT: Activated Partial Thromboplastin Time; CRRT: Continuous Renal Replacement Therapy; LOS: length of stay; RRI: Renal Resistive Index.
