# Supplementary material for: Combined plasma syndecan-1 and renal resistive index as early predictors of sepsis-associated Acute kidney injury: a prospective observational study
Source: Ren Fail. 2026 Feb 18;48(1):2628387. doi: 10.1080/0886022X.2026.2628387 (PMC12918287; doi:10.1080/0886022X.2026.2628387)
Supplement: Supplementary_Table_S1_clean.docx [file IRNF_A_2628387_SM7421.docx]

**Table S1 Demographic and Clinical characteristics for patients with sepsis**

| Variables | Overall  (N = 80) | Sepsis and non-AKI  (N = 39) | Sepsis and AKI  (N = 41) | *P* |
| --- | --- | --- | --- | --- |
| Age | 65 (56-72) | 66 (59-72) | 64 (49-70) | 0.488 |
| Sex |  |  |  |  |
| Female | 27 (33.8) | 18 (46.2) | 9 (22.0) | 0.040 |
| Male | 53 (66.2) | 21 (53.8) | 32 (78.0) |  |
| ICU Severity Scores |  |  |  |  |
| APACHE II | 17 (11.75-23.25) | 15 (10.00-19.50) | 19 (14.00-24.00) | 0.040 |
| SOFA | 5 (3.75-8.00) | 5 (3.00-7.50) | 7 (4.00-9.00) | 0.039 |
| Co-morbidities |  |  |  |  |
| Sepsis shock |  |  |  |  |
| Yes | 18 (22.5) | 6 (15.4) | 12 (29.3) | 0.223 |
| No | 62 (77.5) | 33 (84.6) | 29 (70.7) |  |
| Diabetes |  |  |  |  |
| Yes | 22 (27.5) | 9 (23.1) | 13 (31.7) | 0.539 |
| No | 58 (72.5) | 30 (76.9) | 28 (68.3) |  |
| Hypertension |  |  |  |  |
| Yes | 28 (35.0) | 12 (30.8) | 16 (39.0) | 0.590 |
| No | 52 (65.0) | 27 (69.2) | 25 (61.0) |  |
| Pneumonia |  |  |  |  |
| Yes | 44 (55.0) | 21 (53.8) | 23 (56.1) | 1.000 |
| No | 36 (45.0) | 18 (46.2) | 18 (43.9) |  |
| Perforated Peptic Ulcer |  |  |  |  |
| Yes | 17 (21.2) | 7 (17.9) | 10 (24.4) | 0.667 |
| No | 63 (78.8) | 32 (82.1) | 31 (75.6) |  |
| Atherosclerosis |  |  |  |  |
| Yes | 13 (16.2) | 5 (12.8) | 8 (19.5) | 0.612 |
| No | 67 (83.8) | 34 (87.2) | 33 (80.5) |  |
| Congestive Heart Failure |  |  |  |  |
| Yes | 13 (16.2) | 3 (7.7) | 10 (24.4) | 0.085 |
| No | 67 (83.8) | 36 (92.3) | 31 (75.6) |  |
| Vital signs |  |  |  |  |
| MAP, mmHg | 83(73.75-96.25) | 83 (76.00-94.00) | 85 (73.00-97.00) | 0.870 |
| Heart Rate, beats/min | 96.75 ± 26.52 | 95.33 ± 22.76 | 98.10 ± 29.89 | 0.644 |
| Oxygenation Index, mmHg | 255.00 (187.25-320.00) | 231.00 (181.50-265.50) | 290.00 (193.00-408.00) | 0.019 |
| Laboratory parameters |  |  |  |  |
| Serum Glucose, mg/dL | 8.35 (6.70-10.75) | 8.90 (7.20-11.00) | 8.30 (6.50-10.50) | 0.634 |
| Lactic Acid, mmol/L | 1.50 (1.00-2.40) | 1.40 (0.85-1.90) | 1.70 (1.20-2.70) | 0.024 |
| Creatinine, μmol/L | 102.45 (66.00-158.12) | 66.70 (59.25-92.35) | 158.10 (125.20-195.00) | <0.001 |
| NT-proBNP , pg/mL | 687.90 (315.58-1781.25) | 494.20 (256.70-834.15) | 1207.00 (500.10-4930.00) | 0.001 |
| Albumin, umol/L | 29.68±6.24 | 30.43±5.89 | 28.97±6.55 | 0.297 |
| Total Bilirubin, umol/L | 15.50 (9.88-24.30) | 17.40 (11.10-30.70) | 14.90 (9.00-20.70) | 0.073 |
| BUN, mmol/L | 8.35 (6.47-11.43) | 6.50 (4.40-8.35) | 10.50 (8.30-17.30) | <0.001 |
| Cystatin C, mg/L | 1.20 (0.98-1.70) | 1.00 (0.80-1.10) | 1.60 (1.20-2.10) | <0.001 |
| Homocysteine, μmol/L | 11.80 (7.00-13.95) | 9.50 (6.70-13.00) | 13.20 (9.80-16.30) | 0.005 |
| Prothrombin Time, s | 13.10 (12.20-14.43) | 13.00 (12.35-13.65) | 13.30 (12.00-16.00) | 0.244 |
| APTT, s | 29.20 (26.73-34.05) | 29.00 (26.75-33.90) | 29.40 (26.80-34.30) | 0.874 |
| Serum D-Dimer, mg/L | 4815.00 (1607.50-8900.00) | 4800.00 (1450.00-9460.00) | 5090.00 (1660.00-8570.00) | 0.935 |
| White Blood Cell, 10^9^/L | 11.77±5.86 | 11.78±5.57 | 11.75±6.19 | 0.983 |
| Lymphocytes, 10^9^/L | 0.80 (0.60-1.20) | 0.80 (0.60-1.05) | 0.80 (0.60-1.30) | 0.750 |
| Platelets, 10^9^/L | 183.50 (137.50-255.50) | 202.00 (149.50-235.00) | 180.00 (128.00-266.00) | 0.751 |
| Serum Procalcitonin | 2.34 (0.37-14.80) | 1.06 (0.20-5.42) | 6.50 (0.71-27.50) | 0.003 |
| C-Reactive Protein, mg/L | 69.80 (28.87-113.25) | 62.00 (30.45-113.50) | 71.70 (28.90-113.00) | 0.784 |
| IL-6, pg/mL | 547.50 (109.25-1877.25) | 577.00 (94.60-1095.50) | 518.00 (118.00-3592.00) | 0.508 |
| Serum Amyloid A, mg/L | 141 (81.32-150.00) | 141 (92.65-150.00) | 141(92.65-150.00) | 0.683 |
| Norepinephrine |  |  |  |  |
| Yes | 27 (33.8) | 9 (23.1) | 18 (43.9) | 0.083 |
| No | 53 (66.2) | 30 (76.9) | 23 (56.1) |  |
| Dopamine |  |  |  |  |
| Yes | 18 (22.5) | 11 (28.2) | 7 (17.1) | 0.355 |
| No | 62 (77.5) | 28 (71.8) | 34 (82.9) |  |
| CRRT |  |  |  |  |
| Yes | 9 (11.2) | 0 (0.0) | 9 (22.0) | 0.011 |
| No | 71 (88.8) | 39 (100.0) | 32 (78.0) |  |
| Mechanical Ventilation |  |  |  |  |
| Yes | 46 (57.5) | 21 (53.8) | 25 (61.0) | 0.676 |
| No | 34 (42.5) | 18 (46.2) | 16 (39.0) |  |
| Hospital LOS, days | 16.60（10.52-30.93) | 15.70 (9.15-27.25) | 17.60 (11.80-32.50) | 0.310 |
| ICU LOS, days | 4.85 (2.80-11.75) | 5.00 (2.85-8.55) | 4.80 (2.70-12.30) | 0.851 |
| RRI | 0.65 ± 0.08 | 0.60 ± 0.06 | 0.69 ± 0.08 | <0.001 |
| Syndecan-1, ng/ml | 90.25（65.95-190.73） | 73.67（54.59-109.95） | 109.95（73.67-221.40） | 0.006 |

Continuous variables were expressed as mean ± standard deviation, or median (interquartile range, IQR), as appropriate. Categorical variables were presented as n (%).

**Abbreviations：**AKI: Acute Kidney Injury; APACHE: Acute Physiology and Chronic Health Evaluation; SOFA: Sepsis-related Organ Failure Assessment score; MAP: Mean arterial pressure; NT-proBNP: N-terminal pro-B-type natriuretic peptide; BUN: Blood Urea Nitrogen; APTT: Activated Partial Thromboplastin Time; CRRT: Continuous Renal Replacement Therapy; LOS: length of stay; RRI: Renal Resistive Index.
